# Analysis of influencing factors and construction of nomogram prediction model of postoperative kinesiophobia in patients undergoing total knee arthroplasty

**DOI:** 10.12669/pjms.40.11.10306

**Published:** 2024-12

**Authors:** Jian Xie, Haoliang Cai, Wenting Chen, Xiaohui Wu, Xi Chen, Jun Guo

**Affiliations:** 1 Jian Xie, Dept. of Anesthesiology, Shuguang Hospital Affiliated to Shanghai, University of Traditional Chinese Medicine, Shanghai 201203, P.R. China; 2 Haoliang Cai, Dept. of Anesthesiology, Shuguang Hospital Affiliated to Shanghai, University of Traditional Chinese Medicine, Shanghai 201203, P.R. China; 3 Wenting Chen, Dept. of Anesthesiology, Shuguang Hospital Affiliated to Shanghai, University of Traditional Chinese Medicine, Shanghai 201203, P.R. China; 4 Xiaohui Wu, Dept. of Anesthesiology, Shuguang Hospital Affiliated to Shanghai, University of Traditional Chinese Medicine, Shanghai 201203, P.R. China; 5 Xi Chen, Dept. of Anesthesiology, Shuguang Hospital Affiliated to Shanghai, University of Traditional Chinese Medicine, Shanghai 201203, P.R. China; 6 Jun Guo, Dept. of Anesthesiology, Shuguang Hospital Affiliated to Shanghai, University of Traditional Chinese Medicine, Shanghai 201203, P.R. China

**Keywords:** Total knee arthroplasty, Kinesiophobia, Transcutaneous electrical acupoint stimulation, Influencing factor

## Abstract

**Objective:**

To explore the influencing factors of kinesiophobia and to establish a nomogram prediction model.

**Methods:**

A retrospective observational study was conducted in 354 patients who underwent TKA in Shuguang Hospital Affiliated to Shanghai University of Traditional Chinese Medicine from December 2019 to December 2022. Sociodemographic characteristics, anesthesia methods, Tampa Scale for Kinesiophobia (TSK), numeric rating scale (NRS), hospital for special surgery (HSS), activities of daily living (ADL), and self-rating anxiety scale (SAS) were reviewed and analyzed. Logistic regression analysis was used to identify the influencing factors of kinesiophobia, and a nomogram model was established based on the identified influencing factors and verified by consistency index (C-index), calibration curve, and receiver operating characteristic (ROC) curve.

**Results:**

Incidence of kinesiophobia in patients who received TEAS combined with western medicine (dexmedetomidine and remifentanil) was significantly lower than that in patients who received western medicine alone. The logistic regression analysis showed that family monthly income level, HSS, ADL, and combined TEAS and western medicine approach were independent protective factors for kinesiophobia (all *P*<0.05). The degree of pain and anxiety were independent risk factors for kinesiophobia (both *P*<0.05). A nomogram model was constructed with C-index of 0.721 (0.658, 0.784), and the area under curve (AUC) was 0.748 (95 %CI: 0.685-0.807). The calibration curve also showed good consistency between actual observations and nomogram model predictions.

**Conclusions:**

Family monthly income level, anesthesia methods, NRS, HSS, ADL, and SAS scores are influencing factors of kinesiophobia, and the nomogram model can be useful for predicting postoperative kinesiophobia.

## INTRODUCTION

Knee joint is the most active and weight-bearing flexion joint that is highly susceptible to injury or disease.^1^,^2^ Knee joint diseases, such osteoarthritis that is characterized by pathologic changes in cartilage, bone, synovium, ligament, muscle, and periarticular fat,^3^ are associated with significant pain and affected mobility, seriously impacting quality of life and psychological wellbeing of patients.^4^

In recent years, with the advancement of medical technology, total knee arthroplasty (TKA) has become an important treatment method for knee osteoarthritis.^5^,^6^ However, long term recurrent knee joint pain has a very detrimental effect on the patient’s physical and mental health, often leading to kinesiophobia, an excessive and irrational fear of physical activity or movement caused by increased sensitivity to pain or injury.^7^,^8^ Kinesiophobia is considered a serious factor affecting functional recovery of patients after TKA surgery.^9^ There have been studies on the risk factors for postoperative kinesiophobia in patients undergoing TKA, but there is limited research on prediction model for kinesiophobia after TKA to identify and prevent the risk of kinesiophobia at an early stage.

In addition, there is still a lack of research that specifically focuses on the impact of surgical anesthesia methods on postoperative kinesiophobia in patients undergoing TKA. Clinical studies have confirmed that surgical anesthesia methods have a significant impact on the prognosis of patients.^10^–^12^ Recently, an increasing number of studies have reported the use of transcutaneous electrical acupoint stimulation (TEAS) combined with the standard anesthesia agents, such as dexmedetomidine and remifentanil, in fracture repair and abdominal surgery, and it has been shown to be safe, effective, and with minimal side effects.^11^,^12^ However, there remains a gap of research on the use of TEAS combined with western medicine on postoperative kinesiophobia in patients undergoing TKA.

In this study, we aimed to explore the influencing factors of kinesiophobia after TKA and to establish a nomogram prediction model. Additionally, surgical anesthesia methods (western medicine with or without TEAS) was also studied as an influencing factor.

## METHODS

A retrospective observational study was conducted by retrospectively reviewing the clinical data of patients who underwent TKA surgery in Shuguang Hospital Affiliated to Shanghai University of Traditional Chinese Medicine from December 2019 to December 2022.

### Ethical Approval:

This study was performed in line with the principles of the Helsinki Declaration. The ethics committee of our hospital approved this study, No. 2020-sgys-010, Date: August 24^th^ 2020. The informed consent was waived by the ethics committee for the retrospective nature. The study adhered to the strengthening the reporting of Observational Studies in Epidemiology (STROBE) guideline.^13^

### Inclusion & Exclusion criteria:

Patients underwent TKA surgery, with some oral and written communication skills, and without cognitive impairment were included. Patients with severe cardiovascular, important organ diseases, or mental illnesses such as unclear consciousness and intellectual disabilities were excluded.

### Data collection:

It included general demographic data, anesthesia methods, and multiple evaluation scales.

General demographic data. It included age, gender, BMI, education level, marital status, place of residence, smoking, drinking, hypertension, diabetes, family monthly income, occupation, and medical payment method.

### Anesthesia methods:

Anesthesia methods used were standard western medicine treatment regimen of dexmedetomidine and remifentanil, or a combination of dexmedetomidine and remifentanil with TEAS. TEAS was performed 30 minutes before western medicine anesthesia induction, and the Han’s stimulator was used to stimulate the human acupoints through skin electrodes.

The acupoints were Zusanli and Weizhong, Xuehai and Liangqiu, Yinlingquan and Yanglingquan each in a group, taking the same frequency of electrical stimulation. The stimulation waveform was set to be a sparse waveform of 2-100 Hz, and the intensity was the maximum current intensity (approximately 8-16 mA) that did not cause tingling, numbness, and other discomforts to the patient, and it lasted until the end of the surgical suture.

Western medicine anesthesia was then initiated, and intermittent acupoint electrical stimulation (stimulation for 30 minutes and cessation for 30 minutes) was done during the entire time of the procedure until the end of the surgery.

### Evaluation scales:

To ensure high quality of the study, unified teaching and training for investigators was done before the beginning of the study. The survey was conducted face-to-face with patients, informing them of the purpose and significance of the study, and filling out the precautions part of the survey. Patients were instructed to fill out the survey in a quiet environment. For patients with low educational levels, investigators conducted on-site interpretation and guided patients to truthfully fill out the questionnaire. A total of 360 survey questionnaires were distributed, and 354 valid questionnaires were obtained, with an effective rate of 98.3%. Scales used were as follows:

### Tampa Scale for Kinesiophobia (TSK):

TSK was used for measuring postoperative kinesiophobia. This scale consists of 17 items, with each question using the Likert 4-level scoring method. Higher total score indicates higher level of anxiety, and a score above 37 indicates kinesiophobia disorder.^14^ (2) Numeric rating scale (NRS): NRS was used for pain measurement. It has a score of 0-10, and the pain level is evaluated based on the pain score of zero for “no pain” and 10 for “the worst pain imaginable”, with higher score meaning more severe pain.^15^

### Hospital for Special Surgery (HSS) knee-rating scale:

HSS knee-rating scale was used to evaluate knee joint function. The score has a maximum of 100 points, with higher score corresponds to higher knee joint function.^16^

Activities of Daily Living (ADL): It measures self-care ability through six items (toileting, eating, dressing, continence, indoor moving, and bathing), with the higher score indicative of worse self-care ability.^17^

### Self-Rating Anxiety Scale (SAS):

It contains 20 items and can reflect the patient’s anxiety level: higher score indicates more severe level of anxiety.^18^

### Statistical analysis:

Epidata3.0 software was used to double input the questionnaire. Statistical analysis was conducted using SPSS 22.0 and R software version 4.0.0. Quantitative data were represented by *χ̅*±*S*, and independent sample t-tests were used for inter group comparisons. Count data were represented by n (%), and inter group comparisons were performed using Chi-square test. Logistic regression model was used to analyze the influencing factors of postoperative kinesiophobia. A nomogram model was constructed based on the identified influencing factors using the “rms” package in R software. The consistency index (C-index), calibration curve, and receiver operating characteristic (ROC) curve were conducted to confirm the prediction performance of the model. P<0.05 indicated statistically significant differences.

## RESULTS

A total of 354 patients who underwent TKA were included in this survey. The mean age of the patients was 65.78 ± 7.49 years old. There were 68 males (19.2%) and 286 females (80.8%) in the cohort. Demographic characteristics of patients, such as BMI, education level, history of smoking, history of drinking, marital status, family monthly income, occupation, place of residence, history of hypertension, history of diabetes, medical payment method, and anesthesia method, are summarized in Table-I. The TSK score of the patient was 33.40 ± 9.73 points, with 265 patients (74.9%) scoring ≤ 37 points and 89 patients (25.1%) scoring>37 points. The specific scores of other scales are shown in Table-II.

**Table-I:** Demographic characteristics of patients (n=354).

Item	Classification	n	Composition ratio (%)
Age (year)	<65	152	42.9
	≥65	202	57.1
Gender	Male	68	19.2
	Female	286	80.8
BMI	<24 kg/m^2^	144	40.7
	≥24 kg/m^2^	210	59.3
Educational level	Primary school and below	182	51.4
	Middle and high schools	94	26.6
	College degree or above	78	22.0
History of smoking	No	194	54.8
	Yes	160	45.2
History of drinking	No	194	54.8
	Yes	160	45.2
Marital status	Married	298	84.2
	Unmarried, widowed or divorced	56	15.8
Monthly household income (yuan)	<3000	166	46.9
	3000~5000	107	30.2
	>5000	81	22.9
Career	Active personnel	177	50.0
	Unemployed personnel	114	32.2
	Retired personnel	63	17.8
Place of residence	Rural area	58	16.4
	Town	296	83.6
History of hypertension	No	87	24.6
	Yes	267	75.4
History of diabetes	No	65	18.4
	Yes	289	81.6
Medical payment method	Self-funded	18	5.1
	Urban employee medical insurance	336	94.9
Anesthesia method	Drug anaesthesia	220	62.1
	Integrated Traditional Chinese and Western Medicine Anesthesia	134	37.9

**Table-II:** Patient scores on various scales.

Scale	Score (χ̅±S)
TSK score	33.40±9.73
NRS score	4.04±1.14
HSS score	59.66±5.74
ADL score	71.93±13.10
SAS score	53.02±5.29

Univariate logistic regression showed that there were statistically significant differences in age, monthly family income, anesthesia methods, NRS score, HSS score, ADL score, and SAS score between the groups with and without kinesiophobia (*P*<0.05). There was no statistically significant difference in the comparison of other indicators between the groups (*P*>0.05) (Table-III).

**Table-III:** Comparison of various indicators between groups with and without kinesiophobia.

Item	No kinesiophobia (n=265)	Kinesiophobia (n=89)	χ^2^/t	P
Age (year)			6.392	0.011
<65	124 (46.8)	28 (31.5)		
≥65	141 (53.2)	61 (68.5)		
Gender			0.927	0.336
Male	54 (20.4)	14(15.7)		
Female	211 (79.6)	75 (84.3)		
BMI (kg/m^2^)			0.638	0.424
<24	111 (41.9)	33 (37.1)		
≥24	154 (58.1)	56 (62.9)		
Educational level			1.981	0.371
Primary school and below	132 (49.8)	50 (56.2)		
Middle and high schools	70 (26.4)	24 (27.0)		
College degree or above	63 (23.8)	15 (16.9)		
History of smoking			1.381	0.240
No	150 (56.6)	44 (49.4)		
Yes	115 (43.4)	45 (50.6)		
History of drinking			0.466	0.495
No	148 (55.8)	46 (51.7)		
Yes	117 (44.2)	43 (48.3)		
Marital status			1.733	0.188
Married	227 (85.7)	71 (79.8)		
Unmarried, widowed or divorced	38 (14.3)	18 (20.2)		
Monthly household income (yuan)			9.452	0.009
<3000	116 (43.8)	50 (56.2)		
3000~5000	78 (29.4)	29 (32.6)		
>5000	71 (26.8)	10 (11.2)		
Career			1.207	0.547
Active personnel	133 (50.2)	44 (49.4)		
Unemployed personnel	82 (30.9)	32 (36.0)		
Retired personnel	50 (18.9)	13 (14.6)		
Place of residence			0.730	0.393
Rural area	46 (17.4)	12 (13.5)		
Town	219 (82.6)	77 (86.5)		
History of hypertension			1.215	0.270
No	69 (26.0)	18 (20.2)		
Yes	196 (74.0)	71 (79.8)		
History of diabetes			0.043	0.835
No	48 (18.1)	17 (19.1)		
Yes	217 (81.9)	72 (80.9)		
Medical payment method			1.213	0.271
Self-funded	11 (4.2)	7 (7.9)		
Urban employee medical insurance	254 (95.8)	82 (92.1)		
Anesthesia method			5.990	0.014
Drug anesthesia	155 (58.5)	65 (73.0)		
Combined TEAS and Western Medicine Anesthesia	110 (41.5)	24 (27.0)		
Pain duration (month)	35.51±12.18	37.61±11.93	-1.412	0.159
NRS score (score)	3.94±1.18	4.33±0.97	-2.762	0.006
HSS score (score)	60.61±5.24	56.82±6.24	5.611	0.000
ADL score (score)	72.99±13.30	68.78±12.00	2.650	0.008
SAS score (score)	52.46±4.90	54.66±6.04	-3.107	0.002

Multivariate logistic regression analysis showed that family monthly income, NRS score, HSS score, ADL score, SAS score, and anesthesia method were independent influencing factors for postoperative kinesiophobia (*P*<0.05) (Table-IV). Family monthly income, HSS score, ADL score, and combined TAES and western medicine (dexmedetomidine and remifentanil) were independent protective factors (*P*<0.05), while NRS score and SAS score were independent risk factors (P<0.05). Age was not an independent influencing factor for kinesiophobia (*P*>0.05) (Table-V).

**Table-IV:** Multivariate regression analysis of the influencing factors of kinesiophobia.

Item	B	S.E.	Wald	P	OR	95%CI	

						Lower limit	Upper limit
Age	0.478	0.282	2.887	0.089	1.614	0.929	2.802
Monthly household income	-0.384	0.177	4.689	0.030	0.681	0.482	0.964
Anesthesia method	-0.602	0.291	4.267	0.039	0.548	0.310	0.970
NRS score	0.311	0.123	6.374	0.012	1.365	1.072	1.739
HSS score	-0.103	0.026	15.554	0.000	0.903	0.858	0.950
ADL score	-0.025	0.011	5.437	0.020	0.975	0.955	0.996
SAS score	0.056	0.026	4.567	0.033	1.057	1.005	1.112

**Table-V:** Multivariate regression analysis of the influencing factors of anxiety disorder.

Item	B	S.E.	Wald	P	OR	95%CI	

						Lower limit	Upper limit
Age	0.478	0.282	2.887	0.089	1.614	0.929	2.802
Monthly household income	-0.384	0.177	4.689	0.030	0.681	0.482	0.964
Anesthesia method	-0.602	0.291	4.267	0.039	0.548	0.310	0.970
NRS score	0.311	0.123	6.374	0.012	1.365	1.072	1.739
HSS score	-0.103	0.026	15.554	0.000	0.903	0.858	0.950
ADL score	-0.025	0.011	5.437	0.020	0.975	0.955	0.996
SAS score	0.056	0.026	4.567	0.033	1.057	1.005	1.112

**Fig.1:**
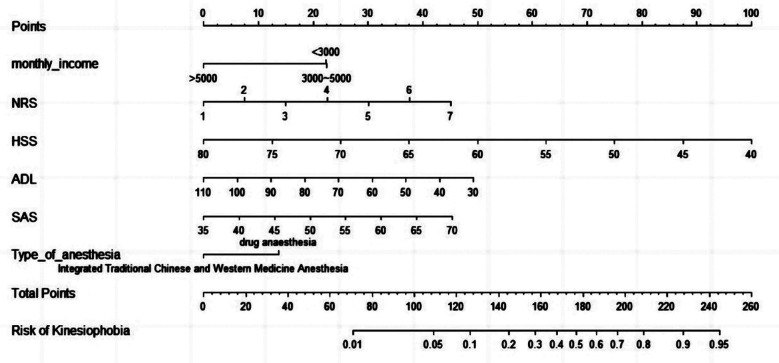
Nomogram prediction model.

**Fig.2:**
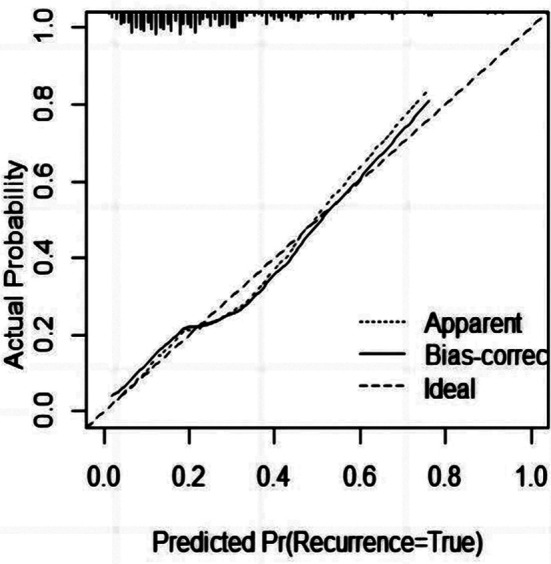
Calibration curve of the nomogram.

**Fig.3:**
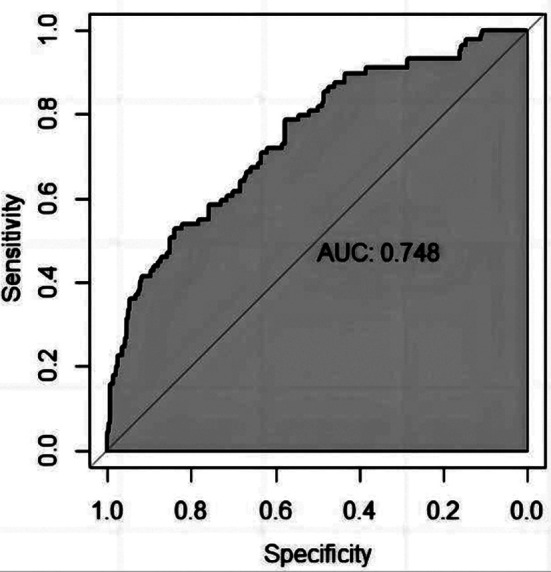
ROC analysis of the nomogram.

A nomogram model was constructed based on the identified independent influencing factors (Fig.1). The nomogram model predicted postoperative kinesiophobia in patients, and the discrimination area under curve (AUC) with 95% confidence interval (CI) of the nomogram model was 0.748 (0.685-0.807), reflecting the good discriminative power of the prediction model (Fig.2). The calibration curve also showed good consistency between actual observations and nomogram model predictions, suggesting that the model had good calibration capability. (Fig.3). Additionally, the C-index was 0.721 (0.658, 0.784). These results verified that the nomogram model had good discrimination and accuracy.

## DISCUSSION

Our results showed that the incidence of postoperative kinesiophobia in patients undergoing TKA in China is relatively high, and the combination of traditional Chinese method (TEAS) and western medicine (dexmedetomidine and remifentanil) anesthesia approach can significantly reduce the incidence of postoperative kinesiophobia in patients undergoing TKA. The occurrence of postoperative kinesiophobia in patients undergoing TKA is mainly influenced by family monthly income level, anesthesia method, pain level, knee joint function, daily self-care ability, and anxiety. This study included 354 patients who underwent TKA surgery. Among them, 89 patients (25.1%) had kinesiophobia, indicating that a quarter of patients are afraid of walking and participating in daily life activities, which is similar to the report by Cai et al.^19^ A systematic review by Brown OS et al.^20^ reported that the incidence of kinesiophobia in patients after TKA was around 19%. Our results indicate that the phenomenon of kinesiophobia after TKA is more prevalent in China compared to the global population, suggesting that medical staff should pay closer attention to such patients.

TEAS regulates the physiological balance of the human body by stimulating the skin at acupoints and regulating the energy of meridians through electrical stimulation.^21^ The study by Yeh ML et al.^22^ confirmed that TEAS combined with dexmedetomidine and remifentanil anesthesia can enhance anesthesia effectiveness, assist in pain relief, reduce the amount of drug anesthesia used, make patients wake up quickly, and reduce side effects that are associated with drug anesthesia. Zhang P et al.^23^ showed that performing TEAS during surgery can alleviate postoperative pain and reduce the incidence of postoperative nausea and vomiting in patients. Our study also confirmed the observations. A recent study by Liu et al also confirms that combining acupuncture therapies with multimodal analgesia can enhance postoperative pain management and improve knee function in patients undergoing TKA, which is similar to our findings.^24^ We may speculate that electrical stimulation reduces primary mechanical hyperalgesia. The reduction of pain hypersensitivity leads to a reduction in activity induced pain, ultimately reducing the occurrence of postoperative kinesiophobia in patients.

Our study also showed that higher monthly family income level was associated with lower likelihood of developing kinesiophobia.^25^ It is plausible that high family income translates into lower overall financial burden, which allows the patient to fully devote themselves to the treatment of the disease and have more energy to learn and implement the requirements of medical staff.^25^

In our study, NRS was identified as an independent risk factor for kinesiophobia, which is consistent with previous studies.^19^,^26^ Due to physical pain, patients develop negative emotions such as fear and avoidance towards physical activities, which are more prominent in patients who experience stronger pain. HSS was identified as an independent protective factor for kinesiophobia, indicating that patients with better knee joint function have a lower incidence kinesiophobia.^27^ We may propose that patients with higher knee joint function have shown significant therapeutic effects after receiving treatment, which increases their confidence in the treatment and overcomes their fear of physical pain. We further show that ADL is also an independent protective factor for kinesiophobia, and patients with high daily self-care abilities are less likely to develop kinesiophobia. SAS was identified as an independent risk factor for kinesiophobia, indicating that patients with greater anxiety are more likely to develop kinesiophobia, which is consistent with previous research findings.^26^,^28^ Severe negative emotions such as anxiety may exacerbate patient’s fear of exercise and pain, leading to incorrect rehabilitation exercise behaviors and reduced daily activities.^29^,^30^ Subsequently, it may lead to even poorer recovery and more severe negative emotions, thus forming a vicious cycle.^31^

We constructed a nomogram model based on identified independent influencing factors, which provides specific scores for each influencing factor. Subsequently, by adding up the scores of each influencing factor, the corresponding probability is obtained. This method transforms complex regression models into a simple graph, making the results of the prediction model more intuitive. The prediction performance of the nomogram model was evaluated, and the results showed that the model had good prediction performance. Clinicians can visually predict the prognosis of patients through nomogram model, and timely develop or adjust reasonable personalized diagnosis and treatment plans.

### Limitations:

Firstly, this is a single center retrospective study. Secondly, the reproducibility and robustness of nomogram model need to be validated in prospective multicenter studies with larger datasets. Further higher quality research is needed to confirm our observations.

## CONCLUSION

The incidence of kinesiophobia in patients undergoing TKA in China is relatively high, and more attention should be paid to it in clinical practice. TEAS combined with western medicine (dexmedetomidine and remifentanil) anesthesia approach is effective in reducing the kinesiophobia rates and may be safely implemented in clinical practice. In addition, more attention should be paid to patients with low monthly income levels, high levels of pain, low knee joint function, low daily self-care ability, and high levels of anxiety. The constructed nomogram model has good predictive performance and can be useful for predicting postoperative kinesiophobia. Medical staff can use this information to identify patients at higher risk of kinesiophobia and take targeted treatment measures in a timely, reasonable, and scientific manner.

### Authors’ contributions:

**JX:** Conceived and designed the study and manuscript writing.

**HC, WC, XW, XC** and **JG:** Collected the data and performed the analysis and critical review.

All authors have read, approved the final manuscript and are responsible for the integrity of the study.
